# Fluorescence Microscopy with Deep UV, Near UV, and Visible Excitation for *In Situ* Detection of Microorganisms

**DOI:** 10.1089/ast.2023.0020

**Published:** 2024-03-19

**Authors:** Noel Case, Nikki Johnston, Jay Nadeau

**Affiliations:** Department of Physics, Portland State University, Portland, Oregon, USA.

**Keywords:** Microscopy, Life detection, Endolithic, Deep UV, Autofluorescence, Fluorescence, Bacteria, Mars analog

## Abstract

We report a simple, inexpensive design of a fluorescence microscope with light-emitting diode (LED) excitation for detection of labeled and unlabeled microorganisms in mineral substrates. The use of deep UV (DUV) excitation with visible emission requires no specialized optics or slides and can be implemented easily and inexpensively using an oblique illumination geometry. DUV excitation (<280 nm) is preferable to near UV (365 nm) for avoidance of mineral autofluorescence. When excited with DUV, unpigmented bacteria show two emission peaks: one in the near UV ∼320 nm, corresponding to proteins, and another peak in the blue to green range, corresponding to flavins and/or reduced nicotinamide adenine dinucleotide (NADH). Many commonly used dyes also show secondary excitation peaks in the DUV, with identical emission spectra and quantum yields as their primary peak. However, DUV fails to excite key biosignature molecules, especially chlorophyll in cyanobacteria. Visible excitation (violet to blue) also results in less mineral autofluorescence than near UV, and most autofluorescence in the minerals seen here is green, so that red dyes and red autofluorescence of chlorophyll and porphyrins are readily distinguished. The pairing of DUV and near UV or visible excitation, with emission across the visible, represents the most thorough approach to detection of labeled and unlabeled bacteria in soil and rock.

## 1. Introduction

### 1.1. Fluorescence microscopy for life detection

It has become increasingly apparent that detection of extant life elsewhere in the solar system will require microscopic imaging techniques; recent reviews cover possible techniques and approaches (Nadeau *et al*., 2018; Enya et al., 2022). Purely chemical techniques [*i.e.,* detection of “substances” (Chan *et al*., 2019)] do not distinguish between extant life and proto-life or abiotic complex chemistry, since we do not have a full understanding of abiotic chemistry or of the early stages of the origin of life (Cleaves, 2012). The addition of detected “objects” to complex chemistry, including micron-scale objects such as cells and microfossils, is an important complement to chemical analysis (Chan *et al*., 2019). However, the design of a microscope for life detection is challenging, involving trade-offs between spatial resolution, volume of view, and speed of throughput.

On Earth, bacterial cells are enumerated by labeling a sample with a dye, usually one that targets nucleic acids (DNA and/or RNA), and cells are individually imaged and counted using fluorescence microscopy with near UV (NUV) to visible excitation (Kepner and Pratt, 1994; Junge *et al.*, 2002; Seo *et al*., 2010). Separating fluorescence of cells from that of the surrounding matrix can be challenging in soil and rock samples. Cells may be within the rock itself (endolithic), or associated with soil particles, biominerals, and other inorganics as well as non-biogenic organic material, all of which may bind dyes (Riis *et al*., 1998; Klauth *et al*., 2004). Organic material may not necessarily be a biosignature; ongoing discoveries of extraterrestrial organics have recently emphasized the importance of understanding abiotic generation of complex organics (McMahon and Cosmidis, 2022). Kerogen-like material of unknown source has been found on Mars as well as on meteorites and is believed to be of abiotic origin (Heinz and Schulze-Makuch, 2020).

Aromatic and aliphatic compounds were released from drilled mudstones in Mars's Gale Crater (Eigenbrode *et al*., 2018), believed to be both indigenous (from abiotic photosynthesis) and exogenous (Franz *et al*., 2020). Mineral fluorescence also presents a formidable background signal (Morono *et al*., 2009). Some pure minerals fluoresce, but the presence and nature of impurities or so-called activators can cause intensity and color of mineral fluorescence to vary from sample to sample (Robbins, 1983; Zhang and Shen, 2023), making it difficult to establish a baseline to subtract. The possibility of label-free detection of bacteria using cellular autofluorescence has been suggested in the astrobiology literature (Bhartia *et al*., 2010), but a systematic comparison of different wavelengths of excitation/emission across the UV to visible has not been performed, even though cellular autofluorescence arises from a variety of molecules. Most methods for bacterial detection on Earth involve dyes or fluorescent proteins. However, there are situations where exogenous probes might be harmful or impractical, such as when imaging is performed directly on a living patient and dyes would pose a risk of toxicity. The challenge of imaging in such a complex environment has provided data of relevance to astrobiology. Label-free fluorescence imaging has been used for diagnosis of infection in wounds (Pijpe *et al.*, 2019; Rennie *et al*., 2019) and for identification of bacterial strain in blood cultures (Walsh *et al*., 2013). In pure cultures, autofluorescence has been shown to be an indicator of resistance phenotype (Costa *et al*., 2020). The difficulty in all these label-free techniques lies in separating the desired autofluorescence from the surrounding tissues or eukaryotic cell(s) (Meyer *et al*., 2020). The quantum yields of native fluorophores are almost always much lower than those of dyes, usually <1%, or the molecules are present at very low concentrations, so spectral separation from background by some method is essential [the exception to this is chlorophyll, which shows emission more intense even than most dyes; it provides useful autofluorescence signatures even in endoliths (Roldan *et al.*, 2014)]. Separation of emission signals might be by color (Dartnell *et al.*, 2010), Stokes shift (Bittel *et al*., 2018), or fluorescence lifetime (Berezin and Achilefu, 2010; Datta *et al*., 2021), which may be measured quantitatively or simply used as a gate to exclude unwanted signals (Shkolyar *et al*., 2018; Yang and Chen, 2020).

### 1.2. Choice of excitation/emission for detecting autofluorescence

**FIG. 1.**
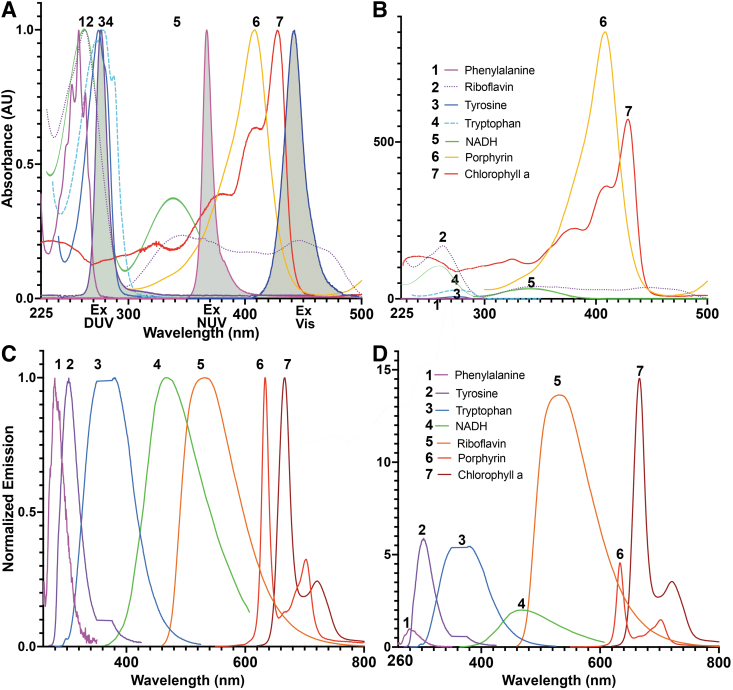
Molecules responsible for microbial autofluorescence. **(A)** Absorbance spectra of key autofluorescent biological molecules. Spectra of the LEDs used for the deep UV, near UV, and visible excitation used in this article are indicated by the gray-shaded curves. For the UV absorbing molecules, peaks at <225 nm were removed to normalize the peaks at ∼280 nm to 1. NADH was normalized to its peak at 250 nm, although it is the peak at 340 nm that is used for imaging. **(B)** Absorbance spectra scaled by the magnitude of the extinction coefficient at the wavelength usually used for excitation. **(C)** Normalized emission spectra of the selected molecules. **(D)** Emission spectra scaled by quantum yield when excited at typical wavelengths (Supplementary Table S1) (Spectra and quantum yields as reported in PhotoChemCad https://www.photochemcad.com/). LED = light-emitting diode; NADH = reduced nicotinamide adenine dinucleotide.

Figure 1 shows absorbance and emission spectra for key molecules involved in microbial autofluorescence (for excitation/emission wavelengths and quantum yields, see Supplementary Table S1). Many datasets do not provide absorbance values below 300–350 nm, so it is important to verify values in the UV when these are of interest.

#### 1.2.1. Deep UV

All cells show autofluorescence from the aromatic amino acids tryptophan, tyrosine, and phenylalanine incorporated into proteins, with excitation peaks at ∼220 and ∼260 nm. It has been suggested that deep UV (DUV) excitation with narrow-band UV emission (excitation 250–260 nm, emission 320 ± 20 nm) presents a “window” in which mineral autofluorescence is minimized but where protein fluorescence may be seen (Bhartia *et al*., 2010). Another study identified 280 nm excitation/340 nm emission in a spectrophotometer as a window for distinguishing bacterial autofluorescence against a background of hospital items (handrails, call buttons, blood pressure cuffs, and disinfectant products); imaging was not performed (Dartnell *et al*., 2013). DUV excitation with UV emission has been poorly explored, largely due to the challenges of building a microscope with components that pass DUV light. Only a few specialized companies carry such instruments, and the objective lenses alone are very costly. Specialized substrates such as quartz or sapphire must also be used due to the fluorescence of glass at these wavelengths. In addition, this excitation will detect any aromatic compound, including kerogen (Shkolyar *et al*., 2018) and contaminants in tap water (Heibati *et al*., 2017).

#### 1.2.2. Near UV

NUV excitation (∼365 nm) will excite tryptophan and tyrosine, though less effectively than DUV; as can be seen in Fig. 1, nearly half of tryptophan's emission occurs in the range 375–500 nm, and both amino acids can be clearly visualized with a standard 4′,6-diamidino-2-phenylindole filter set (Supplementary Fig. S1). NUV will also excite NADH, vitamins such as pyridoxine and riboflavin, and other pigments. Studies have shown that NADH fluorescence dominates in this wavelength range.

The use of reduced nicotinamide adenine dinucleotide (phosphate) [NAD(P)H] as a fluorescent indicator of cell metabolism was first suggested over 60 years ago, and it is currently widely used in cell biology investigations, especially those using fluorescence lifetime imaging microscopy (Li *et al.*, 2008; Szaszak *et al*., 2011; Schaefer *et al.*, 2019) since the lifetime of the molecule shifts drastically when it is bound to proteins. The disadvantage of this wavelength range for excitation in many environmental samples is that it excites nearly all minerals.

#### 1.2.3. Violet to blue

Violet light (405 nm) and blue light (450 nm) excite the Soret band of porphyrins, which show an extremely high peak absorbance in this wavelength range. Emission is in the red, clearly distinguishing these molecules from most other pigments that excite with these wavelength ranges. Of interest to medicine, most pathogenic bacteria contain porphyrins. The red autofluorescence can be distinguished easily from even the most intense tissue fluorescence such as that produced by elastin and collagen, which is green (Konig *et al*., 1998; Ku *et al*., 2019; Rennie *et al*., 2019).

#### 1.2.4. Blue-green

The commonly used 488 nm argon ion laser line, which excites green fluorescent protein (GFP) and fluorescein among many other probes, causes flavin autofluorescence in cells, which can increase when cells are stressed (Surre *et al.*, 2018). This can create unwanted background in imaging and flow cytometry experiments (Yang *et al*., 2012). It is important to note that the emission spectra of cellular autofluorescence by themselves are not a particularly good biosignature. Fluorescence represents electronic transitions that are general to broad classes of molecule. For example, the tryptophan-like peak corresponds to molecules with at least one aromatic ring, which may include amino acids, indoles, polycyclic aromatic hydrocarbons (PAHs), and other compounds both biogenic and abiogenic.

Emission in the blue to green range results from molecules with two or more aromatic rings as well as quinones, flavonoids, and PAHs (Coble *et al*., 2014; Carstea *et al.*, 2020). The value of cellular autofluorescence is in combination with microscopy, where if it creates sufficient contrast with the surrounding substrate so that cell-like objects can be visualized, the morphology of cells and possibly subcellular structures, especially in a repeating pattern, can be highly suggestive of life forms.

### 1.3. Choice of excitation/emission for detecting labeled cells

#### 1.3.1. Deep UV

DUV excitation with visible emission is much easier to implement than when the emission is also in the UV and is rapidly emerging as a technique in biomedicine known as microscopy with ultraviolet surface excitation (MUSE) microscopy (Ho *et al*., 2016; Qorbani *et al*., 2018; Ching-Roa *et al*., 2021). Many dyes show a secondary absorbance peak in the DUV resulting from excitation into the second excited state (S_0_ to S_2_) (Fereidouni *et al*., 2017; Matsumoto *et al.*, 2019). Fluorescent nanoparticles (quantum dots [QDs]) show strong absorbance everywhere below the bandgap and excite well with DUV (Telford *et al*., 2019). The enhanced GFP, commonly used in biological studies for genetically encoded labeling, also has a secondary absorption peak at 280 nm (Dos Santos *et al.*, 2019). The advantages of this wavelength choice are that the excitation light may be delivered externally to the sample at an oblique angle, and no filter is needed to remove it from the signal sent to the camera because of the inability of the associated microscope optics to pass the DUV light. The oblique illumination results in a “shadow” effect revealing sample structure. Disadvantages are the limited power of currently available DUV light-emitting diodes (LEDs), though this is changing rapidly, especially as interest in these wavelengths grows for surface sterilization purposes (Sharma and Demir, 2022).

#### 1.3.2. Visible

Most dyes are engineered for efficient excitation using common laser lines such as argon ion (488 nm) and HeNe (543 nm). Absorbance spectra are broad, so the choice of any blue to turquoise LED will effectively excite most of the common dyes used in bacterial enumeration.

### 1.4. Microscope design considerations

Several fluorescence microscope designs have been proposed or built for space, but often in the context of monitoring bacteria for astronaut health (De Vos *et al.*, 2014). The use of fluorescence microscopy and/or spectroscopy for life detection will not be a viable approach until a rational selection of excitation and emission wavelengths can be chosen based upon realistic designs for labeling and/or autofluorescence detection. In this article, we use a simple robust epifluorescence microscope design to explore the emission spectra of labeled and unlabeled bacteria, minerals, and bacteria-seeded minerals under LED excitation at 275 nm (DUV, oblique illumination), 365 nm (near UV), and 450 nm (visible). Images were collected as wide bandpass signals, with selected bandpass filters, or with a liquid crystal tunable filter (LCTF) to collect wavelength-resolved emission spectra from 420 to 730 nm. Using this instrument, we examined yeast and bacterial cells with and without labeling with the dyes acridine orange, calcofluor white, and QDs. The cells were imaged alone as well as in a mineral context after seeding onto marble. Spectra in solution were also collected in a spectrofluorometer using 275, 365, and 450 nm excitation and cell autofluorescence and dye quantum yields evaluated at these three wavelengths. The relative utility of the different wavelength combinations for cell detection is discussed.

## 2. Methods and Materials

### 2.1. Microscope design

**FIG. 2.**
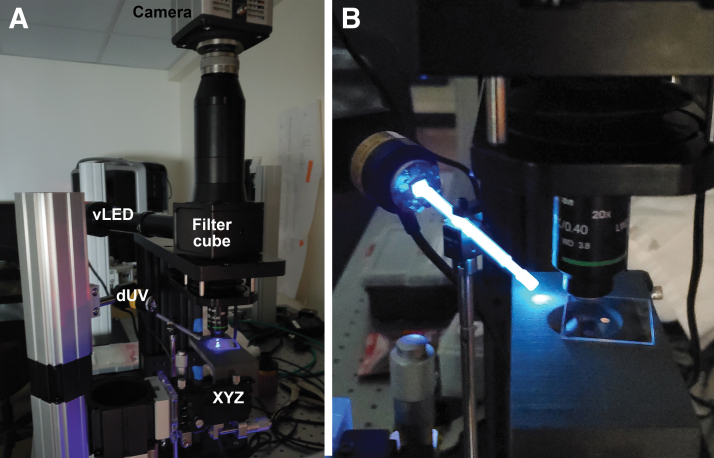
Microscope setup. **(A)** Photo of setup indicating the camera, filter cube, visible or near UV LED (“vLED”), deep UV LED (“dUV”), and manual micrometer stage (“XYZ”). **(B)** Closeup of deep UV illuminator showing the LED coupled to the sapphire rod, causing it to glow. It is not pictured pointing at the sample; for imaging, the brightest spot would be located at the sample area directly under the objective lens.

The microscope used in this study is based upon a modular framework (Cerna with epi-illumination; Thorlabs). We chose an upright rather than inverted design so that excitation light did not have to pass through a slide or other substrate before hitting the sample. For visible excitation, the microscope body (CEA1350) was coupled to an illumination source with conditioning optics, a single filter cube, a tube lens and camera, and a 20 × numerical aperture (NA) = 0.4 or 60 × NA = 0.8 objective lens (Nikon) (Fig. 2A).

Visible excitation was provided by a 450 nm, 2118.1 mW minimum LED (M450LP2; Thorlabs) through an auramine longpass filter (450/50 excitation; 485 nm dichroic; 495 nm longpass emission) (No. 19008; Chroma, Bellows Falls, VT). For near UV excitation a 365 nm, 1150 mW minimum LED (M365LP1; Thorlabs) was used without conditioning optics. The objective lens was a Mitutoyo UV Plan-Apo (20 × NA = 0.5). For near UV and DUV, an “NAR UV” filter set (11004V2; Chroma) was used (365/10 excitation to pass the 365 nm excitation light; 380 nm dichroic; 400 nm longpass emission). To select specific wavelengths of emission, narrow band filters (Chroma and Oriel Instruments) were placed after the filter cube. For DUV excitation, a 275 nm, 45 mW minimum LED (M275L4; Thorlabs) directly illuminated the sample at ∼10° above the plane of the stage. A sapphire rod (SP-25; Swiss Jewel) coupled to a sapphire half-ball lens (48430; Edmund Optics) was used to focus the illumination light onto the sample (Fig. 2B). A 280 nm bandpass filter (53360; Oriel Instruments) was tested in front of the LED to eliminate any possibility of long tail.

White light images were obtained by placing a flashlight on or under the stage for transmitted and oblique illumination, respectively. Full spectra of visible emission were obtained for some samples by using a Kurios multispectral LCTF (KURIOS-WB1, 420–730 nm, 35 nm full width half maximum; Thorlabs). An Andor Zyla 4.2 sCMOS (Oxford Instruments) camera was used for all images. The filter was programmed to trigger upon Andor acquisition using the “trigger out” feature on the Andor camera with a BNC-to-HDB15 cable (RGB-506; Hosa Technology).

Images were acquired in increments of 10 nm using the Micromanager 2.0 software (Edelstein *et al*., 2014), and the resulting image stacks were analyzed in Fiji (Schindelin *et al*., 2012). Illuminant images in HDR format illuminant images were generated in Scyven (Scyllarus, Canberra, Australia) for hyperspectral images. This results in pseudocoloring the emission bands according to a chosen palette; unless stated otherwise, our hyperspectral images correspond to a “real color” RGB palette. Spectral curves of selected regions of interest were generated using the Dynamic Spectrum plug-in for Fiji (https://research.stowers.org/imagejplugins/spectral_imaging.html).

For wavelengths >350 nm, power delivered at the sample was measured using a Thorlabs power meter (PM100D) coupled to a microscope slide power meter sensor head (S170C). For UV, a Coherent Fieldmaster GS with a sensor sensitive to 250–400 nm was used. The area illuminated was measured by calibrating the illuminated spot size on the camera detector with a reticule, or for the case of the sapphire rod illuminator, was simply measured with a millimeter ruler.

### 2.2. Sample preparation

Unless otherwise noted, dyes and culture media were products of Thermo Fisher Scientific (Waltham, MA). Acridine orange was catalog No. A1301, calcofluor white was a component of the Invitrogen Yeast Live/Dead Viability Kit (No. L7009), and cadmium selenide (CdSe)/zinc sulfide core-shell QDs conjugated to streptavidin with emission peak at 585 were catalog No. Q10113MP. QD incubation buffer was catalog No. Q20001MP, and the EZ-Link sulfo-NHS cell surface biotinylation kit was No. A39256. Riboflavin was a product of Alfa Aesar (catalog No. A11764-14), and quinine hemisulfate was a product of Millipore Sigma (catalog No. 22640). Water-soluble, COOH-functionalized cadmium telluride (CdTe) QDs with an emission peak of 605–615 nm were a product of Sigma-Aldrich (No. 777951).

The organisms studied were *Bacillus subtilis* (ATTC 6051; purchased from American Type Culture Collection, Manassas, VA), *Escherichia coli* cloning strain (Invitrogen), *Saccharomyces cerevisiae* or baker's yeast (Red Star Organic Yeast, Milwaukee, WI), *Euglena gracilis* (Carolina Biological Supply, Burlington, NY), and native endolithic communities of *Chroococcidiopsis* present in marble rock samples (gift of Henry Sun; Desert Research Institute, Las Vegas, NV) (Dong et al., 2007). The marble is an opaque white crystalline solid with pockets of green coloration (Supplementary Fig. S3). *B. subtilis* were maintained in lysogeny broth (LB) (catalog No. AAH2676022) and incubated at 37°C for 18–24 h. *Euglena* were maintained in a medium consisting of 12 wheat seeds, rice, a single pea, and 2.5 g milk powder boiled for 10 min in 3 L of spring water; they were stored at room temperature in moderate light. *S. cerevisiae* was grown for 10 min in spring water at 40°C.

All bacterial samples were at least triple washed before and after staining by pelleting in a desktop microcentrifuge, replacement of the medium with ultrapure water, and resuspending. Yeast and *Euglena* samples were not washed. Acridine orange was added to *B. subtilis* at 1 μM final concentration from a stock at 600 μM in water and incubated with rocking for 5–10 min before imaging. *S. cerevisiae* was stained with calcofluor white at a final concentration of 20 μM from a 1 mM stock in water and incubated with rocking at 30°C for 30 min before imaging. All samples were prepared fresh before each experiment. *E. coli* was labeled with streptavidin-QDs by biotinylating the cell surface.

Mid-log cells were pelleted in a microcentrifuge and washed three times with ice cold phosphate-buffered saline. One milliliter of the washed suspension was added to 1 mg EZ-Link “no-weigh” reagent and incubated on ice for 30 min. Cells were then pelleted and washed three times; on the third wash, they were resuspended in QD incubation buffer. Approximately 1 nM streptavidin QDs was added, and the cells were rocked for 60 min before washing three times in distilled water for use.

Marble rocks were crushed to a size such that fragments fit onto glass microscope slides used with the microscope setup but not otherwise processed. Cell viability and staining were confirmed on a standard wide-field fluorescence microscope setup (Olympus IX71) using mercury lamp excitation. Marble was seeded with labeled cells by pipetting 1–5 μL of dyed, washed cells onto the rock surface and allowing to dry completely before imaging.

### 2.3. Absorption and emission spectra and quantum yields

Absorbance spectra were measured using a UV-Visible CLARIOstar plate reader (BMG Labtech) in a UV-Star 96-well plate (Greiner). Fluorescence spectra with visible excitation were collected using the same instrument in epifluorescence mode using a 96-well black plate (Greiner). Emission spectra with DUV excitation were collected on a PC1 photon-counting spectrofluorometer (ISS), which uses xenon arc lamp excitation.

Samples were placed in quartz cuvettes (Starna) for the measurements. Cuvettes were cleaned using a Kontes vacuum cuvette washer (Kimble) connected to house vacuum. They were first rinsed with dilute soapy water, then distilled water, then ethanol, and then finished with distilled water. If contamination was detected in blank runs with ultrapure water, cuvettes were cleaned with sulfuric acid (H_2_SO_4_) before vacu-washing.

Quantum yields were derived by obtaining the slope of the curve of absorbance *A* versus integrated emission *I* and using the formula

(1) $$QY=QY_{ref}\left(\frac{I}{A}\right)\left(\frac{A_{ref}}{I_{ref}}\right),$$

where “ref” refers to the reference fluorophore, which here was fluorescein (for 480 nm excitation) or tryptophan (for 275 nm excitation) or quinine (for 350 nm excitation). All solutions were in water or buffered aqueous solutions with refractive index 1.33, and at least six dilutions were measured for each sample to obtain the linear range of the absorbance curves.

### 2.4. Evaluation of green emission with UV excitation

Because of secondary scatter, emission from turbid solutions could not be evaluated near 2*λ*_ex_. The PC1 instrument minimized secondary scatter by collecting emission perpendicular to excitation so no secondary scatter peaks were observed in clear solutions of pure fluorophores. However, for samples containing cells, secondary scatter made evaluation of green emission from UV excited samples impossible.

## 3. Results

### 3.1. Microscope power at selected illumination wavelengths

#### 3.1.1. Measurements using Thorlabs microscope slide power meter

Microscope power at the sample using the 20 × objective was measured at 330 μW at 450 nm for the visible excitation, 200 μW at 365 nm for the near UV excitation, and 100 μW at 350 nm for the DUV excitation using the sapphire rod and ball lens. The UV objective was essential for use of the 365 nm excitation, as <10 μW at 365 nm was measured through the ordinary glass Nikon objectives, which showed a strong cut-off below 400 nm. The sapphire rod and 275 nm LED tail did not produce measurable power above 400 nm, so no excitation filters were required when the glass objective and/or the hyperspectral filter (lowest wavelength: 420 nm) were used, as confirmed by lack of background signal with up to 30 s of exposure. Background signals at increasing exposure were consistent with leakage of room light and were identical with and without the LED and sapphire rod (Supplementary Fig. S2).

#### 3.1.2. Measurements using Coherent Fieldmaster with 250–400 nm detector

With the 365 nm LED, total power through the objective lens at focus was measured at 200 μW, consistent with the measurement using the Thorlabs sensor; because of the narrow bandpass filter cube, the measurement at 365 nm could be expected to capture essentially all the output power. The 275 nm LED without filters or sapphire rod produced 800 μW in the range 250–400 nm. At the end of the sapphire rod and ball lens, power was 230 μW. The illuminated area at focus was 0.8 mm^2^ for the 20 × objectives and ∼3 mm^2^ for the sapphire rod illuminator.

### 3.2. Absorbance and emission of dyes

**FIG. 3.**
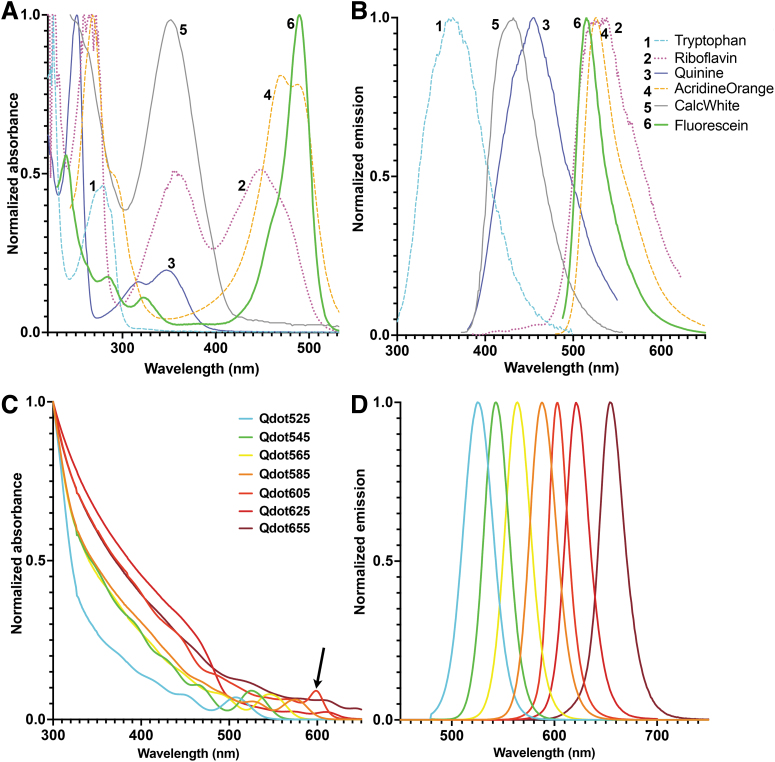
Absorbance and emission spectra of dyes and standards used in this article. **(A)** Absorbance 220–600 nm normalized to the highest peak in the spectrum that occurs within this wavelength range. **(B)** Normalized emission for the molecules in **(A)**. **(C)** Absorbance of a size series of CdSe QDs (Qdot 525 through 655, as reported by Thermo Fisher Scientific). QDs of a given color show a small feature corresponding to the first exciton peak (example at arrow), with strong absorbance at all wavelengths shorter than the peak. **(D)** Emission of the same size series of CdSe QDs, with the radius of the QD determining the color and the size distribution of the particles determining the peak width. CdSe = cadmium selenide; QDs = quantum dots.

Measured normalized absorbance and emission spectra of the dyes and reference standards used are shown in Fig. 3A, B, and measured quantum yields in Table 1. The absorbance peak of tryptophan at 280 nm is significantly lower than the peak that occurs at 230 nm (see Supplementary Fig. S4 for a close-up of the UV region of the spectrum). Of interest is all the tested molecules except fluorescein absorbed strongly at 275 nm. In contrast, only quinine, calcofluor white, and riboflavin absorbed strongly at 365 nm, and only riboflavin, acridine orange, and fluorescein absorbed strongly at 450 nm. A typical absorbance spectrum of CdSe QDs is shown in Fig. 3C, and emission spectra of a size series of CdSe QDs is shown in Fig. 3D. Estimates of quantum yields of QDs are difficult to obtain and have been discussed elsewhere (Grabolle *et al*., 2008; Cao *et al*., 2014); what is important to appreciate from the figure is the high absorptivity everywhere below the excitation peak.

**Table 1.** Measured Quantum Yield of Dyes at Different Excitation Wavelengths

Dye	QY (275 nm excitation)	QY (350 nm excitation)	QY (470 nm excitation)	Relative absorbance (275/peak)
Tryptophan	0.14^a^	Does not excite	Does not excite	1.0
Acridine orange	0.28	ND	0.20	0.86
Calcofluor white	0.26	0.14	Does not excite	1.17
Fluorescein (in 0.1 N NaOH)	0.90	ND	0.91^a^	0.19
Quinine sulfate	ND	0.56^a^	ND	ND
Riboflavin (in 0.1 N NaOH)	0.26	ND	0.26	2.10

Values are in pure aqueous solution, no cell binding.

^a^ Literature reference value for molecule used as standard, from Brouwer (2011).

NaOH = sodium hydroxide; ND = not done; QY = quantum yields.

### 3.3. Autofluorescence

#### 3.3.1. Spectra of culture media

Either disposable plates or quartz cuvettes cleaned with H_2_SO_4_ were essential for UV spectroscopy. Nonspecific organic contaminants on unwashed cuvettes could yield peaks consistent with proteins (excitation peak 280 nm/emission peak 340 nm). It is also important to emphasize that all bacterial images and spectra shown here were taken on cells after repeated washing in distilled water, and either dried on a slide (for images) or resuspended in distilled water (for spectra). In both dried and liquid samples, the growth medium itself as well as the “conditioned medium” left after cells are removed by centrifugation can show intense fluorescence caused by a variety of compounds. For bacteria grown in LB, this fluorescence was different from that of the washed cells. Excitation at 275 nm produced the least background autofluorescence, though a peak in the near UV ∼380 nm was present.

Autofluorescence of the medium was especially intense at 365 nm excitation. At 450 nm excitation, the cells could be seen over the background of the medium, although removal of the medium was still desirable (Supplementary Fig. S5). These background signals can be expected to vary according to the composition of the culture medium, and a full discussion is outside the scope of this article.

#### 3.3.2. Euglena

**FIG. 4.**
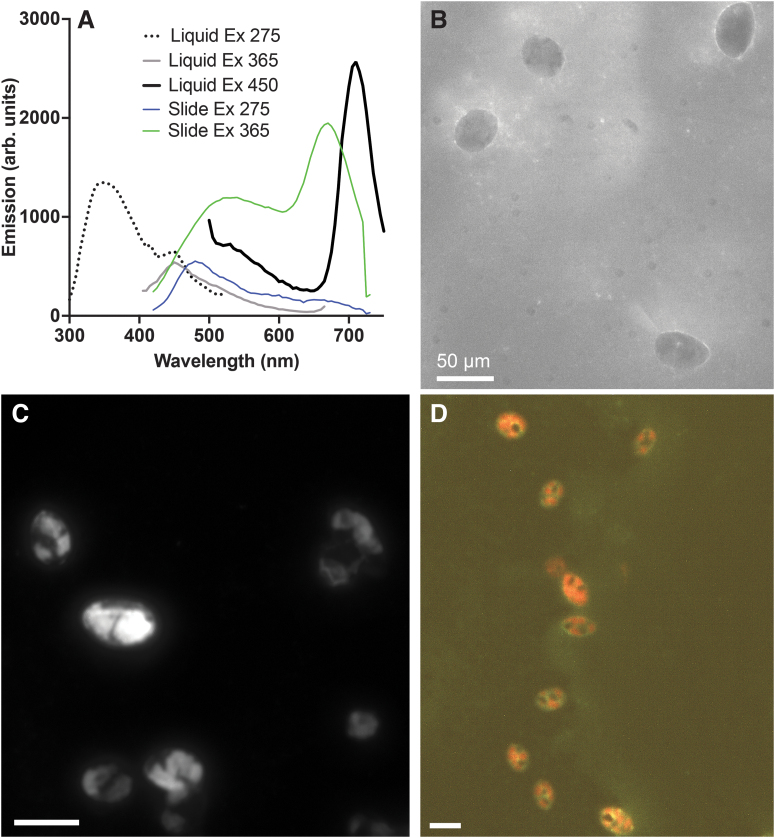
Autofluorescence of a photosynthetic microorganism, *Euglena gracilis*. Peak heights were scaled for ease of interpretation; absolute values of emission peaks are not meaningful. **(A)** Emission spectra with 275, 365, and 450 nm excitation in liquid, and spectra obtained with the same excitation wavelengths on a slide with hyperspectral imaging. Both the visible and near UV excite chlorophyll for imaging. **(B)** 275 nm excitation, 400 longpass emission, 60 × objective, 5000 ms exposure. The chloroplasts appear dark, whereas bright areas indicate other areas of the cell or organic molecules in the medium. **(C)** 450 nm excitation, 500 longpass emission, 60 × objective, 50 ms exposure, region of interest in a chloroplast. The chloroplasts appear extremely bright, swamping any other autofluorescence signature. Their discrete nature can be appreciated in this high-power image. **(D)** 365 nm excitation, 20 × UV objective, hyperspectral image taken 420–730 nm in steps of 10 nm, 1000 ms exposure. The chlorophyll autofluorescence can be seen alongside the broad green autofluorescence signal.

*E. gracilis* is a photosynthetic microalga that serves as an excellent model for cell-based chlorophyll autofluorescence. The emission spectrum of chlorophyll in cells is different from that in solvent and varies among species of microalgae and cyanobacteria (Best et al., 2016; Millie et al., 2002); in *Euglena* in medium using the spectrofluorimeter, we found that the peak was located at ∼710 nm and was optimally excited by violet to blue light. Excitation of washed cells at 450 nm yielded a strong signal and a second, weaker signal in the green (∼535 nm). Excitation at 375 nm yielded a peak at ∼450 nm but did not allow for evaluation of chlorophyll because of secondary scatter at 2*λ*. Excitation at 275 nm showed no chlorophyll signal, but two signals in the UV to blue: one at ∼350 nm corresponding to proteins, and one at 455 nm identical to the one seen with near-UV excitation (Fig. 4A). Imaging under 275 nm excitation showed cells that appeared dark, with a brighter signal coming from fragments of medium (Fig. 4B).

Excitation at 450 nm showed highly fluorescent cells in which the chloroplasts were individually visible (Fig. 4C). Excitation at 365 nm also yielded strong signals from chlorophyll using microscopy (Fig. 4D). Hyperspectral yielded spectra from the specimens on the slides; the curves corresponded to the wavelengths seen in the spectrofluorometer except for the absence of wavelengths below 420 nm. Blue-to-green emission was seen under 275 nm and both blue-to-green and red under 365 nm, whereas the signal at 450 nm was dominated by chlorophyll, especially when sampled in a chloroplast (Fig. 4A).

#### 3.3.3. *S. cerevisiae* and *B. subtilis*

Unpigmented, non-photosynthetic microorganisms, including the yeast *S. cerevisiae* and the bacterium *B. subtilis*, showed similar autofluorescence profiles in the UV (∼340 nm), blue (∼450 nm), and green (∼530 nm) that corresponded to proteins, flavins, and NAD(P)H. The relative intensity of the peaks was highly variable in both organisms; autofluorescence has been shown to change with metabolic state in yeast (Assawajaruwan *et al*., 2017; Maslanka *et al*., 2018) and with stress in bacteria (Surre *et al*., 2018).

**FIG. 5.**
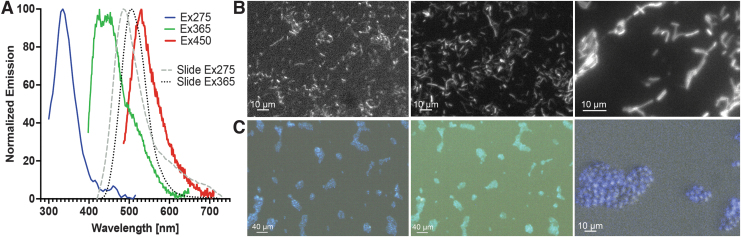
Autofluorescence of *Bacillus subtilis* and *Saccharomyces cerevisiae*. **(A)** Normalized spectra from washed *B. subtilis* cells in solution at 275, 365, and 450 nm excitation. Also shown are spectra collected from slides using the hyperspectral filter at 275 and 365 nm excitation. *S. cerevisiae* showed nearly identical spectra (not shown). **(B)** (Left to right) *B. subtilis*: 275 nm excitation, 400 longpass emission, 2500 ms exposure, 20 × objective; 450 nm excitation, 500 longpass emission, 300 ms exposure, 20 × objective; 450 nm excitation, 500 longpass emission, 200 ms exposure, 60 × objective. **(C**, left**)** *S. cerevisiae*: 275 nm excitation, 1000 ms exposure, 20 × objective, hyperspectral image 420–730 nm in steps of 10. **(C**, center**)** *S. cerevisiae*: 365 nm excitation, 1000 ms exposure, hyperspectral image 420–730 nm in steps of 10, 20 × objective. **(C**, right**)**. *S. cerevisiae*: 4 × zoom of a region from the left panel overlaid with a an oblique white-light image.

Normalized emission spectra with excitation at 275, 365, and 450 nm in liquid as well as spectra obtained on slides with hyperspectral imaging are shown in Fig. 5A. The organisms could be readily visualized using all our excitation wavelengths. Unlabeled *B. subtilis* without a hyperspectral filter with excitation at 275 and 450 nm is shown in Fig. 5B (left, center); a 60 × power image clearly shows the heterogeneity among cells (Fig. 5B, right). Unlabeled *S. cerevisiae* with a hyperspectral filter collecting at 420–730 nm with 275 and 365 nm is shown in Fig. 5C (left, center); a zoom overlay with a white-light image shows the variability of the fluorescence within and among yeast cells (Fig. 5C, right).

### 3.4. QDs and labeled cells

**FIG. 6.**
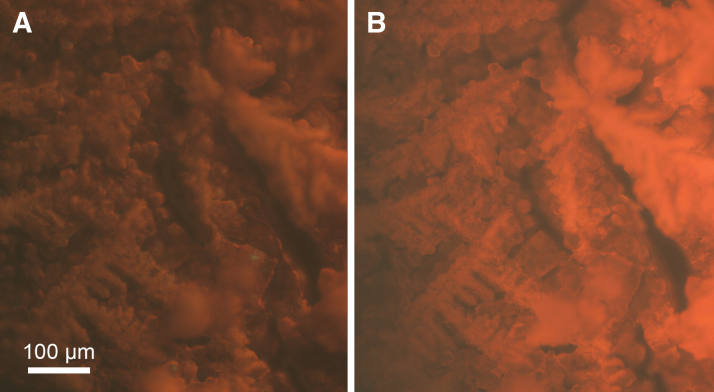
Crystals of CdTe QDs with emission peak at 610 nm. **(A)** 275 nm excitation, 1500 ms exposure. **(B)** 450 nm excitation, 500 ms exposure. Hyperspectral images collected from 470 to 730 nm and RGB pseudocolored.

Both CdSe and CdTe QDs excited with great efficiency at all our tested wavelengths. The oblique illumination of the 275 nm LED provided topographic information (Fig. 6).

Dyes and QDs could be used to label bacterial and yeast cells and excited efficiently with either 275 nm or at their peak (365 nm for calcofluor white; 450 nm for acridine orange). For pure cultures on a slide, the acquisition time could be adjusted to yield the desired signal with or without labeling, and so labeled cells appeared very similar to the label-free images when collected with a longpass filter (Supplementary Fig. S6). The key to the use of labels was the increased signal-to-noise in complex environments and the ability to obtain principal components using hyperspectral imaging, which we explored by imaging cells on marble containing endolithic cyanobacteria.

### 3.5. Endolithic communities with and without seeding

**FIG. 7.**
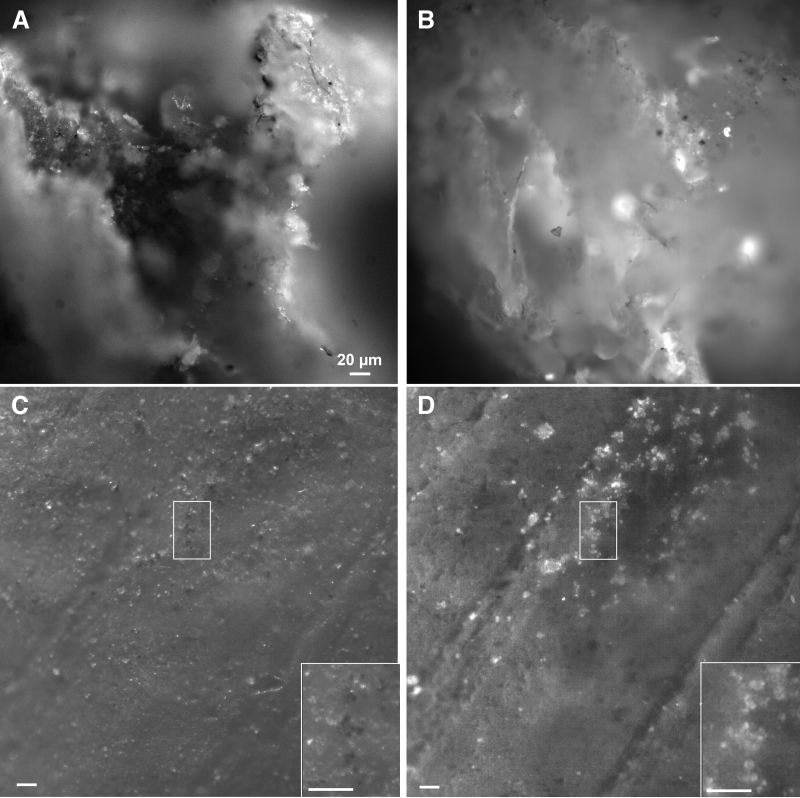
Images of marble containing endolithic cyanobacteria. **(A)** 275 nm excitation, 400 longpass emission, 10,000 ms exposure. **(B)** Same region as **(A)**, 450 nm excitation, 495 longpass emission, 100 ms exposure. The difference in topography resulting from epi- versus oblique illumination can be appreciated in these highly textured samples. **(C)** Rock surface containing cyanobacteria. 275 nm excitation, 420 nm longpass emission, 1000 ms exposure. **(D)** Same region as **(C)**, 365 nm excitation, 420 nm longpass emission, 1000 ms exposure. Insets: 2.5-fold zoom of the same region at each wavelength. Under 365 nm exposure, individual cells could be seen.

The endolithic marble was chosen because it contained clearly distinguishable cyanobacteria and no other significant colonization. Marble fragments containing photosynthetic cyanobacteria appeared very different with 275 nm versus 450 or 365 nm illumination. Under 275 nm excitation, the rock appeared largely dark, though bright filamentous areas of unknown composition were also seen (Fig. 7A). Under 450 nm, bright spots corresponding to photosynthetic organisms could be observed, and the minerals showed a good deal of autofluorescence (Fig. 7B). With excitation at 365 nm, chlorophyll was efficiently excited and individual cyanobacteria could be seen. A comparison of 275 nm excitation and 365 nm excitation (Fig. 7C, D) shows areas of marble with and without cyanobacteria. The DUV excitation resulted in small bright features largely unassociated with the cells; the near UV excitation showed very bright cyanobacteria against a background of mineral autofluorescence.

**FIG. 8.**
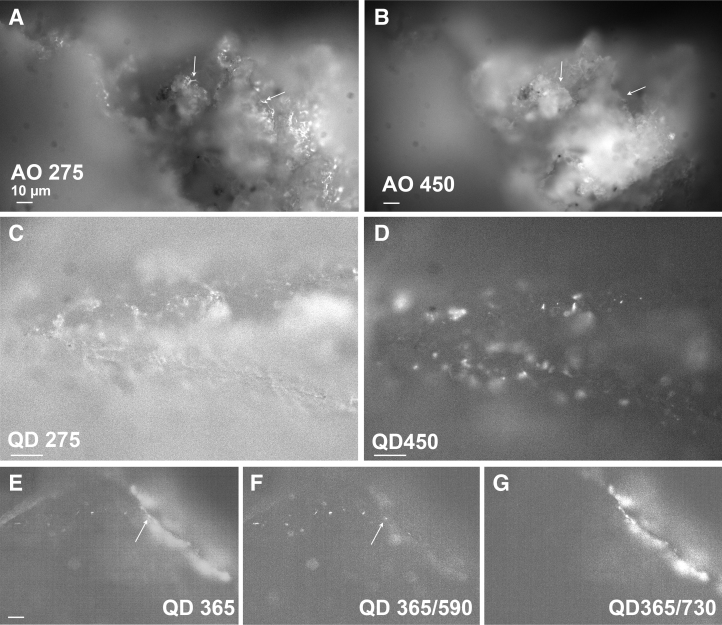
Marble seeded with labeled bacteria. **(A)** Area of rock with acridine-orange labeled *B. subtilis*. 275 nm excitation, 400 nm longpass emission, 1000 ms exposure. The arrows indicate cells as identified by morphology. **(B)** Same region as **(A)**, 450 nm excitation, 495 longpass emission, 100 ms exposure. The arrows show the areas where the cells could be seen in **(A)**, but that are not visible here. **(C)** Area with QD595-*Escherichia coli*, 275 nm excitation, 400 nm longpass emission, 500 ms exposure. **(D)** Same area as **(C)**, 365 nm excitation, 400 nm longpass emission, 500 ms exposure. **(E)** Marble seeded with QD595-*E. coli*, 365 nm excitation, 400 nm longpass emission, 100 ms exposure. **(F)** Same image as **(E)** with a 590/15 nm emission bandpass filter. **(G)** Same image as **(E)** with a 730/15 nm emission bandpass filter.

The marble did not contain unpigmented bacteria, which meant that seeded cells could be confidently known to be the only such cells present. Unlabeled *B. subtilis* seeded onto the rock did not produce sufficient autofluorescence to be reliably detected at any wavelength with monochromatic longpass emission (not shown). When *B. subtilis* was labeled with acridine orange, the cells stood out very clearly against the mineral background with the DUV, but were more difficult to see over the mineral autofluorescence under 450 nm excitation (Fig. 8A, B).

**FIG. 9.**
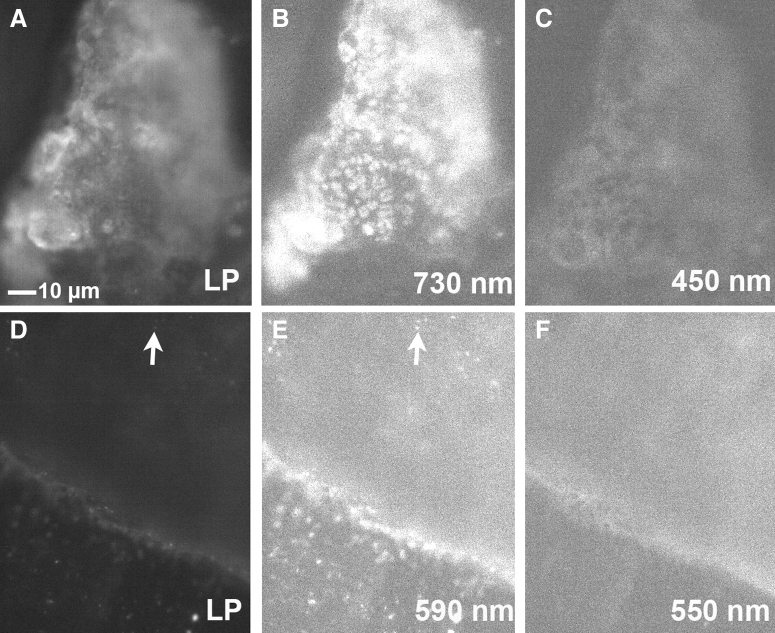
Longpass (LP) versus bandpass filters with photosynthetic and labeled cells. **(A)** Region of marble under 420 nm longpass. **(B)** Same region as **(A)** with a 730/15 bandpass filter. **(C)** Same region as **(A)** with a 450/15 bandpass filter. **(D)** Region of marble seeded with *E. coli*-QD585 under 420 nm longpass. **(E)** Same region as **(D)** with a 590/15 bandpass filter. Note that some cells that are difficult to see under the longpass appear clearly in the bandpass image (example at arrow); many of the cells are visible in both images as can be seen in the lower portion of the figures. **(F)** Same region as **(D)** with a 550/15 bandpass filter, showing complete absence of the QD signal at this wavelength.

QD-labeled bacteria were easily seen at all our excitation wavelengths (Fig. 8C–G). There were advantages and disadvantages to each wavelength. Greater overall background was seen in the QD sample with 275 nm exposure, perhaps reflecting some amount of QD material remaining in the cell suspension even after washing (Fig. 8C). Excitation at 450 nm provided the least background and greatest signal to noise in longpass grayscale images (Fig. 8D). With 365 nm exposure, the cyanobacteria were maximally excited, and distinguishing the signal from the QDs was difficult without separation of the signals with bandpass filters (Fig. 8E–G). The use of bandpass filters aided in disambiguating nearly all signals. Longpass filters provided only contrast and morphological information, which were difficult to interpret when organisms were near the resolution limit of the instrument. Narrow-band filters could be used to confirm the identity of an autofluorescent molecule or of a dye or other label. This was particularly useful if the target signature had a characteristic wavelength that was outside most mineral autofluorescence, such as chlorophyll (Fig. 9A, B), or if it had a narrow emission peak such as QDs (Fig. 9C, D).

### 3.6. Hyperspectral images

**FIG. 10.**
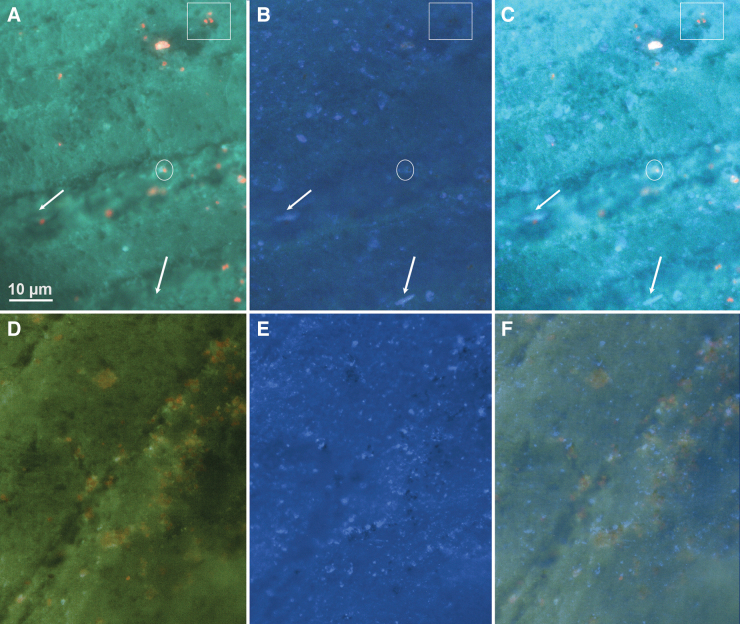
Detection of unlabeled *B. subtilis* on marble. **(A)** NUV (365 nm) excitation with emission 420–730 in steps of 10 nm. **(B)** DUV (275 nm) excitation with emission 420–730 in steps of 10 nm. **(C)** Image with both LEDs illuminated at full power. The unlabeled bacteria could be seen only with 275 nm excitation (arrows). Photosynthetic cells (red, example in rectangle) appeared dark under 275 nm excitation, but many of these cells were accompanied or outlined by material apparent in the DUV image (example in ellipse). **(D)** Control sample with NUV excitation containing only cyanobacteria. **(E)** Control sample with 275 nm excitation. **(F)** Control sample with both NUV and DUV excitation. DUV = deep UV; NUV = near UV.

The use of a hyperspectral filter allowed for separation of mineral autofluorescence and biosignatures either with color alone or using spectral analysis. Capturing of emission in spectral bands and pseudocoloring removed much of the ambiguity in the samples containing unlabeled bacteria. Figure 10 shows a comparison of NUV and DUV excitation of a region of marble containing cyanobacteria and seeded with unlabeled *B. subtilis*. In the NUV image (Fig. 10A), the photosynthetic cyanobacteria were clearly visible, but the *B. subtilis* were not apparent. In the DUV image (Fig. 10B), the chlorophyll could not be seen, but several *B. subtilis* cells were apparent from their morphology. However, nonspecific smaller areas with the same spectrum were also seen, probably reflecting organics. With both LEDs illuminated (Fig. 10C), it was possible to see both cell types. Unseeded marble samples are shown as controls in Fig. 10D–F, illustrating the presence of the smaller DUV-excited areas of fluorescence that were sometimes but not always associated with cyanobacteria.

**FIG. 11.**
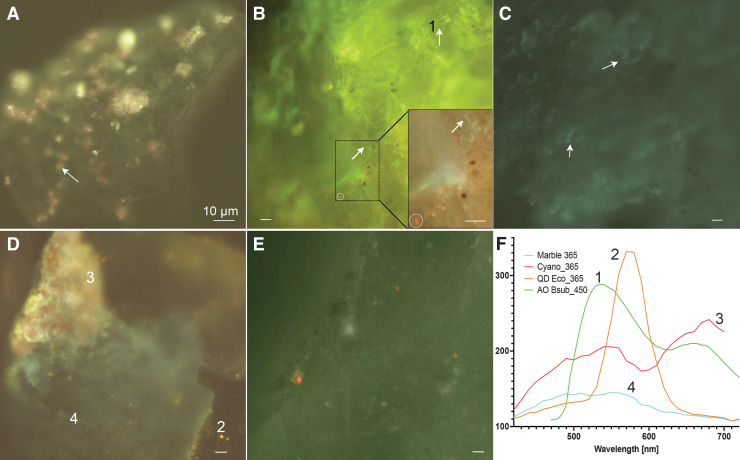
Hyperspectral images of Santa Barbara marble with and without addition of labeled bacteria. Images are pseudocolor palettes applied to image stacks taken every 10 nm from 470 to 730 nm (for 450 nm excitation) or 420–730 nm (for 365 and 275 nm excitation). **(A)** Unlabeled marble at 450 nm excitation, showing cyanobacteria emitting a characteristic red fluorescence (example at arrow) over a background of nonspecific green. **(B)** Marble seeded with *B. subtilis* labeled with acridine orange, 450 nm emission. Cells can be seen over the background at the arrows; a cyanobacterium is circled. The inset shows a 2 × zoom of the selected region with principal component analysis. The *B. subtilis* cells are clearly visible in the supper right corner (arrow) as is the cyanobacterium in the lower left (circle). **(C)** Marble with acridine orange-labeled *B. subtilis* under 275 nm excitation. Cells can be seen at arrows. **(D)** Marble with QD 595-labeled *E. coli*, 365 nm excitation. Cyanobacteria on the edge of the imaged rock face appear red, whereas QDs appear orange. **(E)** Marble with QD 595-labeled *E. coli*, 275 nm excitation. QD-labeled cells appear orange. **(F)** Small region of interest (∼10 × 10 μm) spectra collected from the areas labeled 1–4. 1: *B. subtilis* with acridine orange, 450 nm excitation, from panel **(B)**. 2: *E. coli* labeled with QD595, 365 nm excitation, from panel **(D)**. 3: Cyanobacteria, 365 nm excitation, from panel **(D)**. 4: Background area of rock in QD-labeled area, 365 nm excitation, from panel **(D)**.

Hyperspectral analysis permitted separation of more complex signals resulting from NUV and visible excitation. In unlabeled mineral samples, autofluorescence gave a broad green peak, whereas cyanobacteria gave a signal centered at 680 nm (Fig. 11A, F). With acridine orange labeling, although the spectrum overlapped the autofluorescence, pixel-by-pixel analysis of cell-like objects showed characteristic dye spectra, and principal component analysis could be used to enhance contrast (Fig. 11B, inset). Under 275 nm illumination, the emission spectrum was essentially flat across the emission range, and multispectral analysis was less valuable than for visible excitation (Fig. 11C). Acridine orange represented an example of one of the most challenging dyes to separate from mineral autofluorescence. Calcofluor white showed significantly less overlap and could be readily distinguished on minerals (not shown). QDs may be chosen at any emission wavelength; red and orange were also very easy to detect. The near UV (365 nm) excitation wavelength produced the most useful images containing both orange QDs (595 nm emission) and chlorophyll. Both were readily excited at this wavelength and had comparable emission intensities. The spectra were readily distinguished (Fig. 11D, F). Under 275 nm excitation, the QD-labeled cells were also readily seen, although the contrast was less than with the near UV or visible, consistent with what was seen with the long-pass images (Fig. 11E).

### 3.7. Transillumination versus oblique illumination

For transparent samples, the DUV LED could be placed under a quartz slide for transillumination. In this case, a 275 short pass or bandpass filter was essential to exclude the tail of the LED illumination. This arrangement did not provide the topographical features seen with oblique illumination but was convenient and simple to set up. Its disadvantages are the requirement for a bandpass filter to eliminate the excitation tail, and the inability to image opaque samples. Supplementary Fig. S7 shows a comparison of a QD sample illuminated from below versus at an angle with 275 nm.

## 4. Discussion

There are a few reports of fieldable fluorescence microscopes, and even fewer developed for space flight. One key reason for this is the difficulty of building portable, vibration- and shock-resistant illumination sources. LEDs have advantages over lasers and arc lamps in terms of cost, safety, and ruggedness. LED technology has improved tremendously over the past 10 years, now permitting excitation down into the DUV. We first reported a field-worthy LED-based fluorescence microscope for life detection in 2010; at the time, the shortest wavelength LED reasonably available was “Royal Blue” (450 nm) (Rogers *et al*., 2010). Here, we report an updated low cost, LED-based microscope with visible, near UV, and DUV excitation and visible emission. The addition of these wavelengths produces some substantial differences in biosignature detection.

Currently available DUV LEDs emit much less power than those emitting in the near UV or visible. However, this is rapidly changing. The fact that DUV is eye safe because of its short penetration depth, and its ability to inactivate pathogens such as coronavirus (Gerchman *et al*., 2020), has led to tremendous recent development in LED technology in this wavelength range. This, along with increasing interest in techniques such as MUSE microscopy, means that the current limitations on DUV excitation can be expected to disappear quickly. Even using the relatively weak DUV sources readily available as of 2022, the strengths and weaknesses of excitation with this wavelength range can be appreciated. The advantages of DUV are the ability to excite many fluorophores with a single wavelength and a large effective Stokes shift. We confirm here that the quantum yields of common dyes (fluorescein, acridine orange, and calcofluor white) are the same with 275 nm excitation than with near UV or visible excitation. This is an expected result, as it shows consistency with Kasha's rule; however, exceptions to this rule do exist (del Valle and Catalán, 2019) and some results on this subject have been contradictory, largely because of the difficulty of directly comparing results from different spectrometers. Despite using different instruments for the visible/NUV and DUV ranges, we found very consistent quantum yield results for the different excitation peaks. Semiconductor QDs, both CdSe and CdTe, also excite extremely well in the DUV.

Another feature of DUV that may be an advantage or disadvantage is the fact that it does not pass through traditional optics. This means that collecting UV emission is difficult, and excitation is easiest when delivered directly as opposed to through a filter cube. DUV filters with high transmissivity have been difficult to obtain, though they are available from several suppliers currently. On the other hand, filters are not needed to eliminate stray excitation light after the sample if objectives that do not pass UV are used. However, because of the long tail on LED emissions, a short pass or bandpass filter immediately after the source is often needed, especially if transillumination is used.

Another useful feature of the DUV is its ability to efficient excite flavins and NAD(P)H even more effectively than visible light. Flavins and NAD(P)H significantly contribute to the autofluorescence spectrum of unpigmented bacteria, and autofluorescence of numerous strains has been reported to overlap that of flavins; such autofluorescence increases under cell stress (Surre *et al*., 2018). While much has been made of protein (tryptophan) autofluorescence, most of this signal is not captured by traditional optics since the emission peaks in the UV. However, the flavin signal is entirely visible. In traditional microscopy and flow cytometry, bacterial flavin autofluorescence is excited with an argon ion laser (488 nm), and the stronger absorbance peak in the DUV has been disregarded or considered as noise rather than signal. However, the photophysics of this transition has been examined (Ghosh and Puranik, 2018), and there is emerging interest in cellular autofluorescence as a useful signal (Jamme *et al*., 2013; You *et al*., 2018; Boppart *et al*., 2019; Morrow and Elowsky, 2019; Coronado-Parra *et al*., 2022; Tian 2022). The use of DUV to excite green bacterial autofluorescence may prove useful in environmental and biomedical applications. An issue for spectroscopy is that both flavins and NADH emit close to 2*λ* when excited with DUV. This makes collection of absorbance spectra tricky since secondary scattering in many spectrometer setups completely obscures this signal. The same is true for green dyes and proteins such as acridine orange or GFP.

Finally, DUV excites less undesirable mineral autofluorescence and LB medium autofluorescence than near UV but does excite some degree of blue fluorescence. Due to the association of this signal surrounding cyanobacteria, we suspect that this signal is from nonspecific organic material, though it could also represent mineral autofluorescence. Mineral and medium autofluorescence swamp the signals of unlabeled cells with 365 and 450 nm excitation, as found here. The fluorescence seen on the minerals with both 275 and 450 nm excitation likely represents organic compounds, as can be appreciated in the hyperspectral images of Figs. 9 and 10, where some of the cyanobacteria can be seen to be accompanied or outlined by blue-to-green-fluorescent material. However, this was not universal; many of the photosynthetic cells appeared dark under DUV illumination. Spots with similar spectra occurred elsewhere on the rock as well, and it was unknown whether these were organic molecules or mineral fluorescence, which can show similar peaks (Shkolyar *et al.*, 2021). Regardless of the source, this emission range is nonspecific as it indicates essentially any organic matter; depending on the experiment, organic but non-living material might be signal or noise.

We deliberately chose a test sample with no native unpigmented organisms for this preliminary work, so that we would be certain that these signals were not bacteria until bacteria were seeded. Because of this background, identifying unpigmented seeded bacteria on rocks by autofluorescence alone was difficult, though it was possible as shown in Fig. 10, and may be useful as a first-pass approach to identifying areas of interest for further downstream analysis by other techniques. One key disadvantage of DUV excitation is its inability to excite chlorophyll, at least in the intact organisms used here. Chlorophyll is probably the single most useful fluorescent biosignature on Earth, at both the microscopic and planetary level (Kiang *et al*., 2007b). In field applications such as the Imaging Flow Cytobot (Olson and Sosik, 2007), only chlorophyll-containing organisms are imaged since this is the only signal strong enough and distinctive enough to separate cells from debris.

It is likely that molecules with similar spectral features will have evolved elsewhere; porphyrins have been suggested to be an ideal biomarker (Suo *et al*., 2007), and fluorescence of chlorophylls largely follows that model (Gouterman, 1961). The absorbance peaks of chlorophylls relative to the emission of different spectral classes of stars have been discussed and may be similar to Earth's even around different classes of stars (Kiang *et al*., 2007a; Mehta *et al*., 2014; Komatsu *et al*., 2015). The red fluorescence of chlorophyll and porphyrins (600+ nm) is also distinct from mineral and medium autofluorescence, which is green (500–550 nm). It is important to use quartz rather than borosilicate glass slides for studies of red fluorescence, since glass will emit in this range (Yan *et al*., 2021).

The dark appearance of the photosynthetic microorganisms we observed in this study was novel and interesting, and to our knowledge has not been previously shown. Most studies do not report the spectral properties of chlorophyll below its Soret band (peak at ∼400 nm). A few spectroscopic (not imaging) studies have extended into the DUV (Persichetti *et al*., 2019), including at least one using synchrotron radiation (Cerovic *et al*., 2002). While excitation of chlorophyll is possible in the 275–280 range, UV is highly damaging to chlorophyll, and most photosynthetic organisms have evolved UV-protective pigments to protect it (Cerovic *et al*., 2002). In plant samples, the presence of these pigments can be quantified by comparing the efficiency of chlorophyll excitation with UV versus green light (Bilger *et al*., 2001), where the attenuation of the emission with UV excitation indicates shielding. The endolithic organisms in the gypsum and marble studied here, *Chroococcidiopsis*, was collected in the Mojave desert; desert *Chroococcidiopsis* is known to have extremely high levels of UV protective pigments (Casero *et al*., 2021), and cyanobacteria in general produce a variety of screening pigments such as mycosporine-like amino acids and scytonemins, which absorb strongly throughout the UV range (Rastogi and Incharoensakdi, 2014; Häder and Rastogi, 2021). The nearly black appearance of the cyanobacteria observed here under UV imaging likely reflects this shielding and will be explored further in future work.

Specific labels greatly enhance the ease, specificity, and certitude of microbial detection in complex environments. Dyes that target very general classes of biosignatures such as lipids and nucleic acids are available and can be expected to work on any water-based world. A review of this topic has been published recently (Enya *et al.*, 2022). The dyes tested here have also been shown to withstand the radiation environments of missions (Thompson *et al*., 2006; Carr *et al*., 2013; Hanczarek *et al*., 2018). Labels add brightness, narrow emission bands allowing for principal component analysis, and chemical specificity. Dyes can also help show cell structure. It can be appreciated from Figs. 4 and 5 that autofluorescence emission is not uniform across the cells and may not provide an outline that clearly shows cell shape or size. Either brightfield overlays or membrane-targeting dyes can greatly assist with morphological biosignatures. Although morphology alone is unlikely to be sufficient for definitive detection, especially for microfossils (Callefo *et al*., 2019), it can be highly suggestive and serve as a useful pre-screening technique to identify potentially interesting samples. The fluorescence microscope being developed for a Mars mission by the Japanese Space Agency uses a panel of dyes to detect cells (Enya *et al*., 2022).

One of the typical disadvantages of dyes is the need for multiple excitation wavelengths. The use of the secondary UV excitation peaks to yield emissions spanning the visible makes traditional dyes more convenient and potentially simplifies instrument design. Specific QD probes are also of potential use, although targeting biosignatures with functionalized QDs is more difficult. Research into the optimal panel of dyes for a life-detection mission remains to be performed and will need to be optimized for each planetary target. With any autofluorescence or dye selection, some kind of spectral information is important to be able to distinguish cells from substrate. With only long-pass emission, even the brightest pigments or labels only provide brightness contrast. The use of bandpass, or hyperspectral filters can be used to create RGB or pseudo-colored images that distinguish specific emission features. Principal component analysis allows for extraction of specific spectra even when they strongly overlap autofluorescence signals.

## 5. Conclusion

DUV plus near UV and/or visible LED excitation with visible emission represents a simple, low-cost approach to fluorescence microscopy that is potentially useful for life detection, but the addition of specific labels greatly facilitates identification of cells on rocks. Hyperspectral imaging or multi-bandpass filters can increase specificity and contrast.

## Supplementary Material

Supplemental data

Supplemental data

Supplemental data

Supplemental data

Supplemental data

Supplemental data

Supplemental data

Supplemental data

## Notes

Associate Editor: Victor Parro

## Abbreviations Used

CdSe: cadmium selenide
CdTe: cadmium telluride
DUV: deep UV
GFP: green fluorescent protein
H_2_SO_4_: sulfuric acid
LB: lysogeny broth
LCTF: liquid crystal tunable filter
LED: light-emitting diode
MUSE: microscopy with ultraviolet surface excitation
NA: numerical aperture
NADH: reduced nicotinamide adenine dinucleotide
NAD(P)H: reduced nicotinamide adenine dinucleotide (phosphate)
NUV: near UV
PAHs: polycyclic aromatic hydrocarbons
QDs: quantum dots
