# Supplementary material for: Fluorescence Microscopy with Deep UV, Near UV, and Visible Excitation for In Situ Detection of Microorganisms
Source: Astrobiology. 2024 Mar 19;24(3):300–17. doi: 10.1089/ast.2023.0020 (PMC10979697; doi:10.1089/ast.2023.0020)
Supplement: Supplemental data [file Suppl_TableS1.pdf]

Molecule	Excitation used for QY measurement (nm)	Emission peak (nm)	Quantum yield	Molar extinction coefficient (M^{-1} cm^{-1})
[1]Phenylalanine	240	280	.022	195
[1]Tyrosine	260	304	.13	1405
[1]Tryptophan	270	355	.12	5579
[1]Riboflavin (in ethanol)	450	530	.3	33000
[2]NADH	340	460	Varies with conditions in cells	
[1]Protoporphyrin IX (in chloroform)	407	633	.06	171000
[1]Chlorophyll a (in methanol)	418	670	.32	111700

Table S1. Photophysical parameters of biomolecules as reported in spectral databases: [1]PhotochemCAD; [2] See: Ma et al., Biomed. Opt. Express, 2016 Jul 1; 7(7): 2441–2452.
