# Supplementary material for: Fluorescence Microscopy with Deep UV, Near UV, and Visible Excitation for In Situ Detection of Microorganisms
Source: Astrobiology. 2024 Mar 19;24(3):300–17. doi: 10.1089/ast.2023.0020 (PMC10979697; doi:10.1089/ast.2023.0020)
Supplement: Supplemental data [file Suppl_FigS1.pdf]

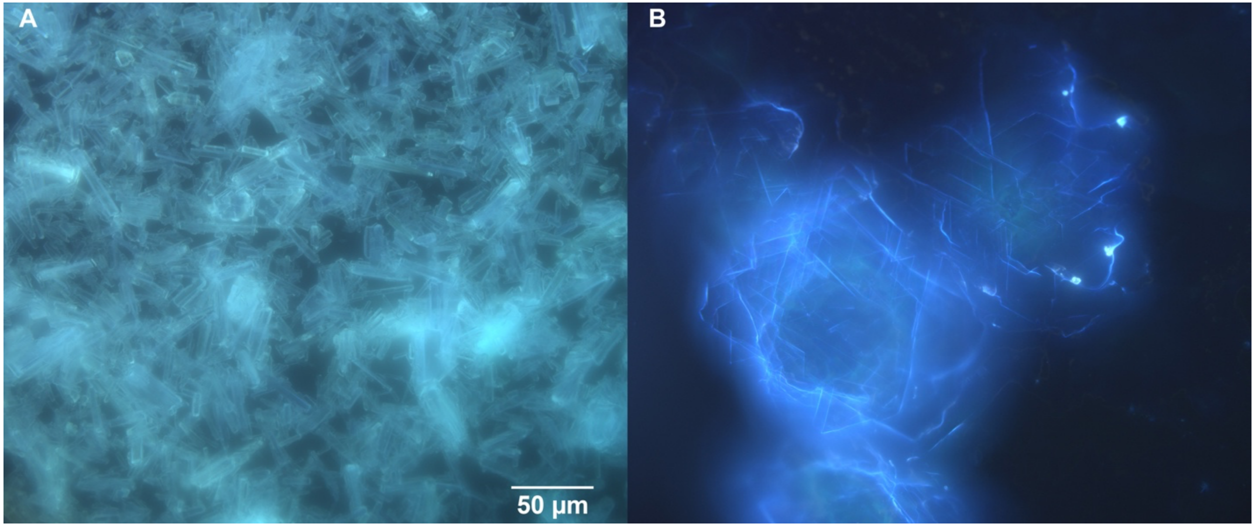

Figure S1. Images of (A) tyrosine and (B) tryptophan crystals on an Hg lamp-powered epifluorescence microscope with an RGB camera (Zeiss Axiocam), excitation 375-400 nm and emission 418-448 nm.
