# Supplementary material for: Fluorescence Microscopy with Deep UV, Near UV, and Visible Excitation for In Situ Detection of Microorganisms
Source: Astrobiology. 2024 Mar 19;24(3):300–17. doi: 10.1089/ast.2023.0020 (PMC10979697; doi:10.1089/ast.2023.0020)
Supplement: Supplemental data [file Suppl_FigS2.pdf]

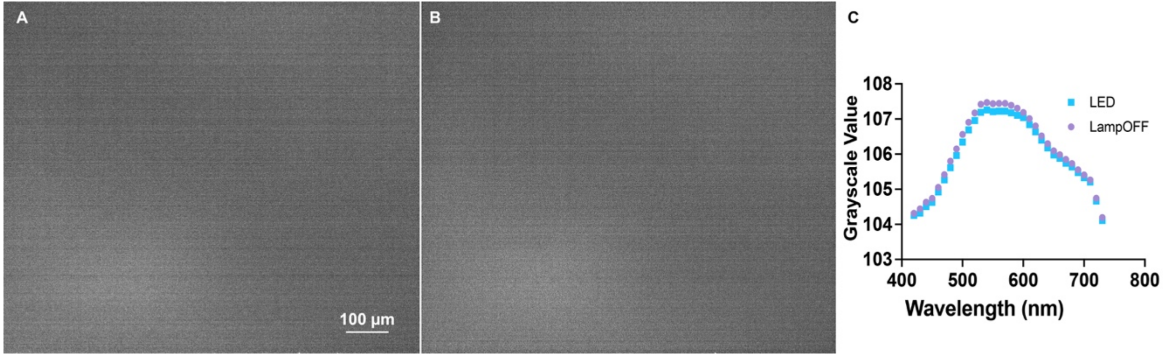

Figure S2. Background signals with 30 s exposure times. (A) Longpass background with illumination off. (B) Longpass background with 275 nm LED and sapphire rod, no sample. (C) Spectra of background showing signal peaking at 550 nm with and without the LED, consistent with room light.
