# Supplementary material for: Fluorescence Microscopy with Deep UV, Near UV, and Visible Excitation for In Situ Detection of Microorganisms
Source: Astrobiology. 2024 Mar 19;24(3):300–17. doi: 10.1089/ast.2023.0020 (PMC10979697; doi:10.1089/ast.2023.0020)
Supplement: Supplemental data [file Suppl_FigS4.pdf]

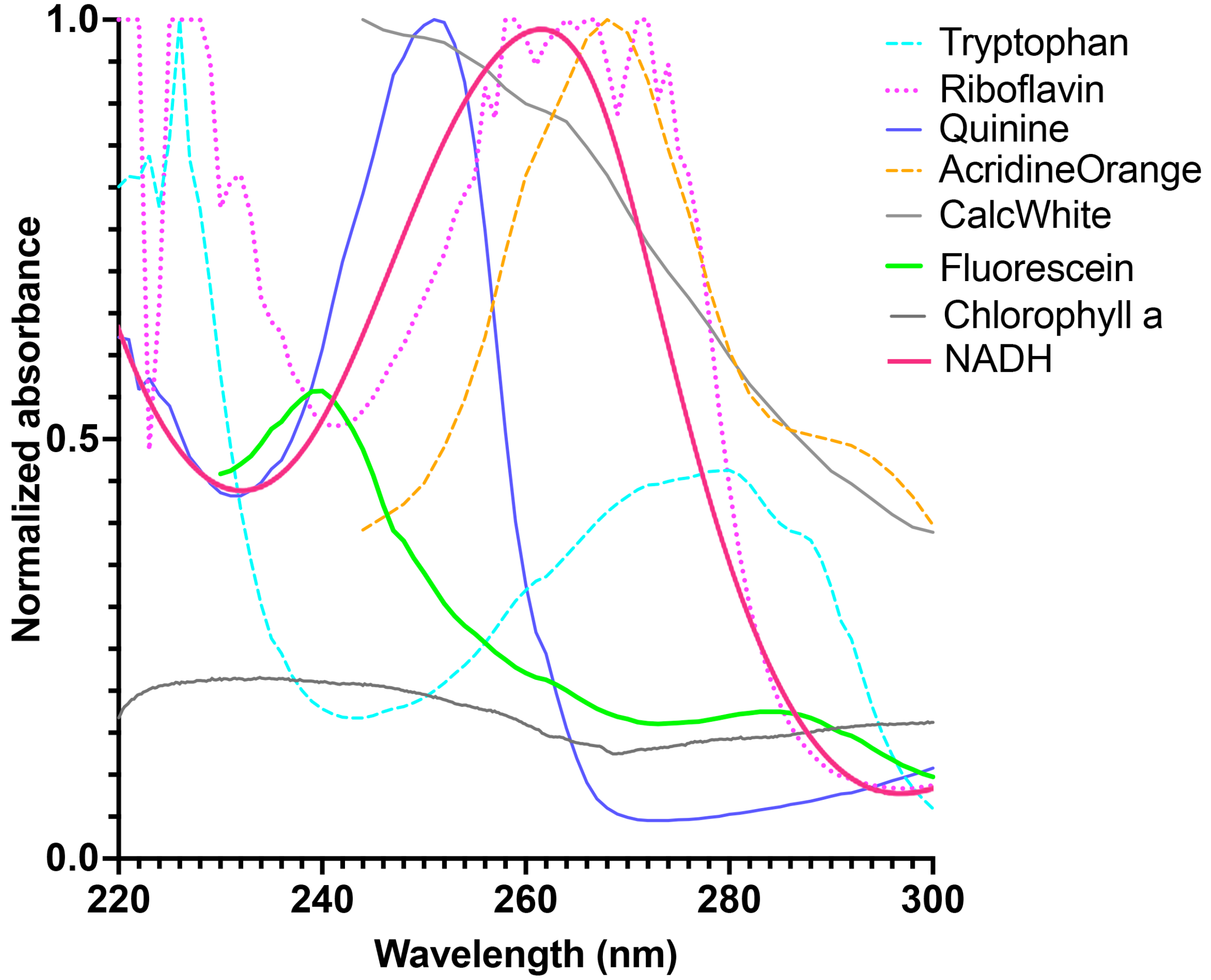

Figure S4. UV (225-300 nm) absorption spectra of dyes and autofluorescent biomolecules normalized to the highest peak in this region. Many public databases omit this region, and most organic solvents absorb below cutoff wavelengths in the UV (examples: acetone, 329 nm; benzene, 278 nm; dimethylformamide, 267 nm) (reference for cutoffs: https://chem.libretexts.org/Bookshelves/Analytical_Chemistry/Physical_Methods_in_Chemistry_and_Nano_Science_(Barron)/04%3A_Chemical_Speciation/4.04%3A_UV-Visible_Spectroscopy).
