# Supplementary material for: Fluorescence Microscopy with Deep UV, Near UV, and Visible Excitation for In Situ Detection of Microorganisms
Source: Astrobiology. 2024 Mar 19;24(3):300–17. doi: 10.1089/ast.2023.0020 (PMC10979697; doi:10.1089/ast.2023.0020)
Supplement: Supplemental data [file Suppl_FigS5.pdf]

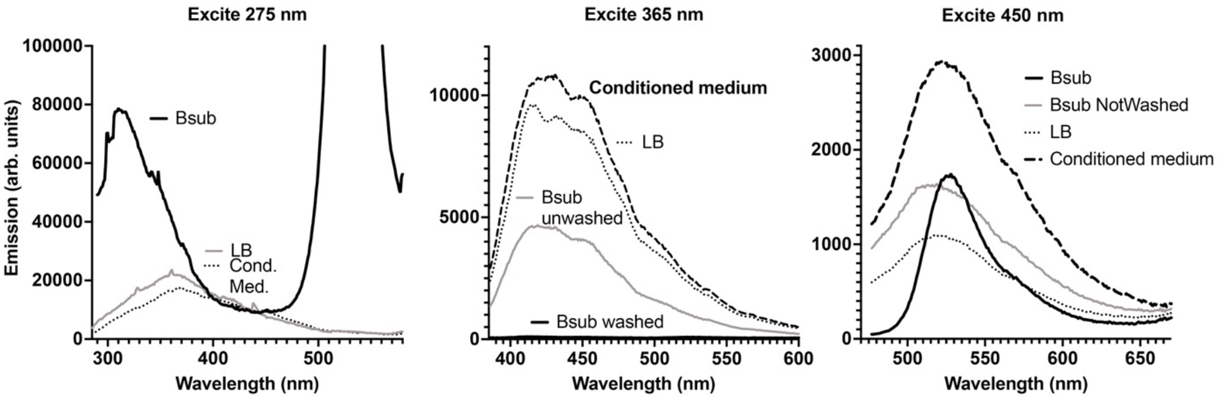

Figure S5. Emission spectra of LB culture medium, "conditioned medium" recovered after cell growth by pelleting cells, and unwashed *B. subtilis* compared with the washed cells. The emission scale on the 275 nm excitation is deliberately reduced to show the UV emission peaks; the green peak is scattering.
