# Supplementary material for: Fluorescence Microscopy with Deep UV, Near UV, and Visible Excitation for In Situ Detection of Microorganisms
Source: Astrobiology. 2024 Mar 19;24(3):300–17. doi: 10.1089/ast.2023.0020 (PMC10979697; doi:10.1089/ast.2023.0020)
Supplement: Supplemental data [file Suppl_FigS6.pdf]

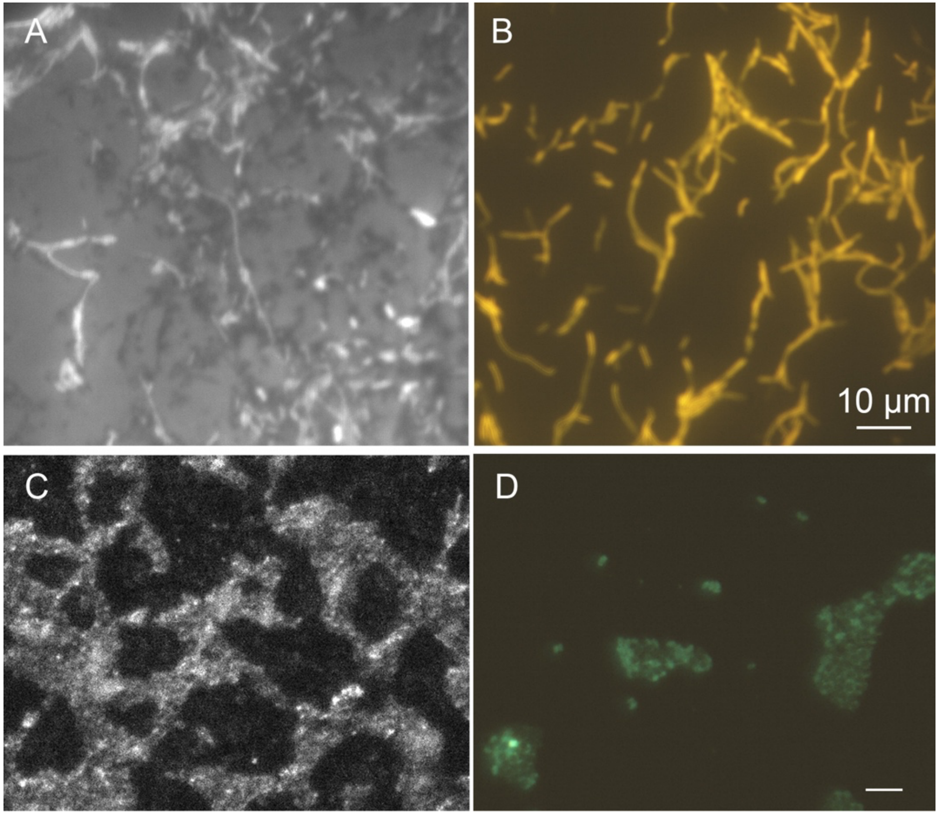

Figure S6. Dye-labeled cells. (A) *B. subtilis*, 60x, acridine orange, 275 nm excitation, 500 ms exposure. (B) *B. subtilis*, 60 x, acridine orange, 450 nm excitation, 200 ms exposure (with hyperspectral imaging). (C) *S. cerevisiae*, 20x, calcofluor white, 275 nm excitation, 100 ms exposure. (D) *S. cerevisiae*, 20x, calcofluor white, 365 nm excitation, 400 ms exposure (with hyperspectral imaging).
