# Supplementary material for: Fluorescence Microscopy with Deep UV, Near UV, and Visible Excitation for In Situ Detection of Microorganisms
Source: Astrobiology. 2024 Mar 19;24(3):300–17. doi: 10.1089/ast.2023.0020 (PMC10979697; doi:10.1089/ast.2023.0020)
Supplement: Supplemental data [file Suppl_FigS7.pdf]

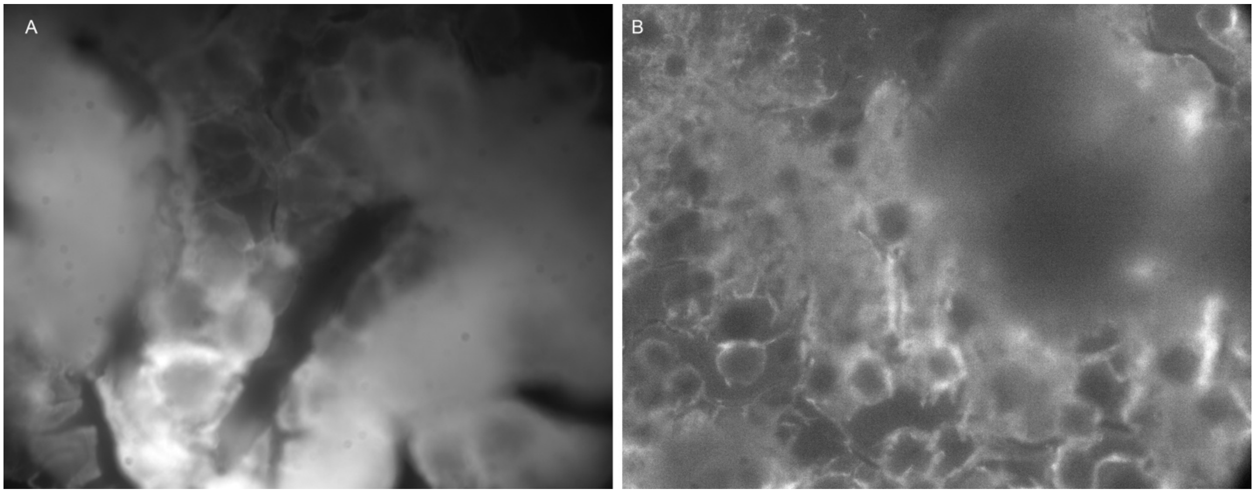

Figure S7. Transillumination vs. oblique illumination. Image of CdTe QD crystals on a quartz slide illuminated (A) from below and (B) obliquely.
